# Gynostemma Pentaphyllum Increases Exercise Performance and Alters Mitochondrial Respiration and AMPK in Healthy Males

**DOI:** 10.3390/nu15224721

**Published:** 2023-11-08

**Authors:** Deepti Nayyar, Xu Yan, Guoqin Xu, Min Shi, Andrew P. Garnham, Michael L. Mathai, Andrew J. McAinch

**Affiliations:** 1 Institute for Health and Sport, Victoria University, P.O. Box 14428, Melbourne, VIC 8001, Australia; deepti.nayyar@live.vu.edu.au (D.N.); sean.yan@vu.edu.au (X.Y.); shimin0816@gmail.com (M.S.); andrew.garnham@deakin.edu.au (A.P.G.); michael.mathai@vu.edu.au (M.L.M.); 2 Australian Institute for Musculoskeletal Science (AIMSS), Victoria University, P.O. Box 14428, Melbourne, VIC 8001, Australia; 3 College of Exercise and Health, Guangzhou Sport University, Guangzhou 510500, China; xugq@gzsport.edu.cn

**Keywords:** adiponectin, blood glucose, leptin, oxygen flux

## Abstract

This research aimed to determine the effects of Gynostemma pentaphyllum (*G. pentaphyllum*) on exercise performance, AMP-activated protein kinase (AMPK), and mitochondrial signaling in human muscle. This randomized double-blind placebo control crossover study provided placebo or 450 mg of *G. pentaphyllum* dried leaf extract equivalent to 2.25 g of dry leaf per day for four weeks to 16 healthy untrained young males, separated by four weeks wash-out. Following 4-week supplementation with *G. pentaphyllum,* participants had significantly lower leptin and blood glucose levels and improved time trial performance over 20 km, which corresponded with a higher muscle oxygen flux compared to placebo. Muscle AMPK Thr172 phosphorylation significantly increased after 60 min exercise following *G. pentaphyllum* supplementation. AMPK Thr172 phosphorylation levels relative to total AMPK increased earlier following exercise with *G. pentaphyllum* compared to placebo. Total ACC-α was lower following *G. pentaphyllum* supplementation compared to placebo. While further research is warranted, *G. pentaphyllum* supplementation improved exercise performance in healthy untrained males, which corresponded with improved mitochondrial respiration, altered AMPK and ACC, and decreased plasma leptin and glucose levels.

## 1. Introduction

Gynostemma pentaphyllum (*G. pentaphyllum*) is a creeping perennial herb from the Cucurbitacea (melon/gourd) family with a characteristic 5-pointed leaf. The fruit is considered inedible, and only the leaves and stems are used. It grows in temperate climates commonly found in South and East Asia, such as Thailand, Malaysia, the Philippines, China, India, Japan, and Korea [1]. *G. pentaphyllum*, also known as jiaogulan, is an herb that has been used as a food and supplemental food product for more than 500 years [2]. *G. pentaphyllum* is also consumed as a tea brewed from the dried leaves. No harmful effects have been reported, and toxicity testing in rats demonstrated that the plant extract does not cause mortality or organ damage at a high dose of 1000 mg/kg/day for 90 days [3]. *G. pentaphyllum* contains many bioactive components including saponin, flavonoids, and sterols [4]. Due to these bioactive compounds, *G. pentaphyllum* is suggested to be beneficial in the treatment of various health conditions, such as obesity [5], hyperlipidemia [6], inflammation [7], and tumor suppression [6]. While the effects of *G. pentaphyllum* have recently been reviewed extensively [8], 12 weeks of supplementation with *G. pentaphyllum* significantly reduced total abdominal fat area (−20.90 ± 8.29 cm^2^), subcutaneous fat (−8.70 ± 3.54 cm^2^), and waist circumference (−2.49 ± 0.35) [5]. Furthermore, 16 weeks of *G. pentaphyllum* supplementation significantly reduced fat mass by 1.2% [9]. In addition to these human studies, *G. pentaphyllum* reduces body weight and fat in obese mice by significantly enhancing energy utilization, and also reduces the level of total cholesterol and low-density lipoproteins [10]. Furthermore, it was also found that *G. pentaphyllum* decreased the size and total weight of white adipocytes and decreases body fat in mice [11].

In addition to the beneficial effects on reducing cholesterol and fat, saponins of *G. pentaphyllum* also increased the phosphorylation of AMP-activated protein kinase (AMPK) in L6 myotube cells [12] and enhanced glucose uptake [13]. AMPK is an intracellular energy sensor that regulates metabolic homoeostasis and is therefore a target for various metabolic syndromes, such as obesity and diabetes [14]. AMPK enhances ATP production and is activated during adenosine triphosphate (ATP) shortage, heat stress, excessive training, hypoxia, and starvation [15]. The activation of AMPK was demonstrated to increase exercise performance in mice [16]. Mitochondria are a primary source of ATP production in the cell. Therefore, any reduction in ATP synthesis in mitochondria during resting and stress conditions may induce AMPK activation [17]. It was observed in rats under energy stress that AMPK was activated, thus increasing mitochondrial biogenesis in skeletal muscles [18]. Therefore, any substance that activates AMPK may act as an exercise simulant to enhance mitochondrial biogenesis in muscles [16]. Mitochondrial biogenesis is a complex process that also involves multiple transcriptional factors, including peroxisome-proliferator-activated receptor gamma coactivator 1-alpha (PGC-1α) [19]. Besides the positive impact on AMPK and mitochondria in cellular models, no study has investigated the effects of *G. pentaphyllum* on muscle AMPK expression and mitochondrial function in healthy humans. Therefore, the current study aimed to evaluate the effects of *G. pentaphyllum* supplementation on exercise performance, AMPK activity and signaling, and mitochondrial function in healthy males.

## 2. Materials and Methods

### 2.1. Study Design

**Figure 1:**
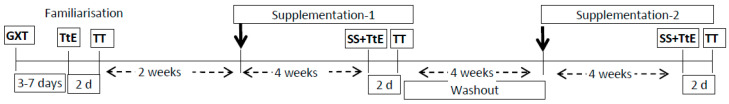
Trial design. GXT = graded exercise test to determine VO_2_peak, ventilatory threshold, and corresponding power outputs; TtE = time to exhaustion at power output 50% between Ventilatory Threshold and VO_2_peak; TT = 20 km time trial; SS = steady state cycling: 60 min at 90% of ventilatory threshold, 5 min of active recovery.

This research was approved by the Human Ethics Research committee of Victoria University, Melbourne, Australia (HRE17-151). Written informed consent was obtained from all participants involved in the study. Nineteen healthy untrained males (aged 18–35 years) with BMIs less than 25 kg/m^2^ were recruited via social media and sporting organizations to participate in this randomized crossover, placebo-controlled, double-blinded supplementation trial (Figure 1). Four weeks of supplementation was considered sufficient to determine changes in AMPK activity in skeletal muscle, as this time period and number of participants have been utilized in other supplementation and exercise studies respectively [20,21]. Participants were excluded if they had any known cardiovascular or respiratory conditions (e.g., asthma, cardiac arrhythmias), hypertension (resting blood pressure > 145/95 mm Hg), bleeding disorders, eating disorders, skin or anesthetic allergies, musculoskeletal injuries that may be aggravated by the exercise protocol, type 1 or 2 diabetes, or current medication including: anti-hypertensives, insulin sensitizers, anti-obesity drugs, and steroidal medications. Physical activity status was confirmed via the Australian physical activity questionnaire [22], which was completed following baseline testing. Sixteen out of nineteen participants completed the intervention. Three of the participants dropped out, one prior to the first testing day following 4 weeks of supplementation and two following the first supplementation period and testing, citing difficulty with time commitment to continue with the study. Data from the three participants who dropped out were excluded from all analyses.

The effects of the supplementation with *G. pentaphyllum* or placebo on aerobic exercise performance were completed using two different measures, including steady-state and time to exhaustion (TtE) tests and a 20 km time trial (TT). All testing on participants was completed at the exercise physiology laboratory at Victoria University. Participants first undertook a graded exercise test (GXT) to determine their peak aerobic power, which was then used to set the exercise intensities for the steady-state submaximal exercise (SS) and TtE tests.

### 2.2. Graded Exercise Test (GXT) and Familiarization

Peak aerobic power (VO_2peak_) was assessed during a symptom-limited GXT on a cycle-ergometer (Excalibur sport, Lode B. V., Groningen, The Netherlands). The protocol consisted of an initial intensity of 60 W, then an increase of 30 W every 2 min. The test was terminated when a participant’s rating of perceived exertion (RPE) reached “very hard” (Borg scale = 17) or before that if the patient wished to stop or clinical signs or symptoms of metabolic or cardiorespiratory abnormalities appeared. Expired respiratory gases were collected through a breath-by-breath (BxB) pneumotach system connected to gas analyzers (Cosmed Quark CPET, COSMED, Rome, Italy). The BxB data were integrated over each 15 s interval, and the mean values for oxygen consumption (VO_2_), carbon dioxide generation (VCO_2_), and ventilation (VE) were calculated for each interval. The gas analyzers were calibrated immediately before each test as per the manufacturer’s instructions.

Dietary intake was controlled for the 3 days prior to the initial GXT via the provision of all food for the participants. Energy requirements for each participant were estimated using the Schofield equation and adjusted for physical activity level, and food intake was tailored to the client’s likes/dislikes such that an energy balanced diet was consumed that met the Australian guide to Healthy Eating (Foodworks, Version 10, Xyris Software, Brisbane, Australia 2019). Diets were designed by a qualified dietitian.

A familiarization of the TtE and TT within a week of the GXT was undertaken to ensure exercise intensities estimated from the GXT were appropriate and allowed the participants to become familiar with the exercise tasks to reduce daily test–retest variability between trials. During the familiarization of the SS and TtE, participants cycled at the SS power output determined by the GXT to elicit an intensity of 90% of ventilatory threshold for 10 min to verify that the power output was indeed below the ventilatory threshold. The work rate was then increased to a power output at 50% of the ventilatory threshold and the VO_2_ peak until exhaustion, which was then repeated following 30 min of rest. Two days following the SS and TtE familiarization session, participants undertook one familiarization of the 20 km TT. The participants were familiarized with the distance of the time trial and the Velotron™ cycle ergometers (SRAM LLC, Chicago, IL, USA). This familiarization has previously been shown to be sufficient to lower variability from 3% to less than 1% [23].

Two weeks following the familiarization testing, the participants started with either placebo or the *G. pentaphyllum* supplement (randomized and double blinded) for 4 weeks and then undertook the SS + TtE and TT, separated by two days (Figure 1). The participants then completed a four-week washout period before repeating the supplementation and exercise testing regimen with the alternative supplement (i.e., placebo or *G. pentaphyllum*) not used in the first trial (Figure 1).

### 2.3. Supplementation

Two weeks after the 20 km TT familiarization, participants were provided with 60 capsules of either placebo or the *G. pentaphyllum* supplement. Participants were asked to consume two capsules each day with their morning meal. Both placebo and *G. pentaphyllum* product with the brand name ACTIVAMP were made into capsules by Your Solutions compounding pharmacy (Brisbane, Australia), complied with Therapeutic Goods Australia guidelines, and were identical in shape, color, and taste when consumed whole as instructed. Each placebo capsule contained 500 mg of maltodextrin, and each *G. pentaphyllum* capsule contained 225 mg of *G. pentaphyllum* dried leaf extract, equivalent to 1.125 g of dry leaf plus 275 mg of maltodextrin, within a ~22 mm length capsule. Dosage of *G. pentaphyllum* was based on previously published protocols [9]. Maltodextrin is generally regarded as safe, and the dosage used was very low compared to levels that can cause side effects [24]. Participants did not report any side-effects when consuming the placebo and *G. pentaphyllum* capsules. The *G. pentaphyllum* extract used in the capsules was processed according to the method described previously [25]. Briefly, the dried leaves were extracted with 5:1 purified water and ethanol and dried to a powder. The extract powder used in the capsules was prepared from a single batch of the plant extract. Batch analysis confirmed that the levels of active compounds were consistent with previous extracts: 20(S)-Ginsenoside Rg3 (0.24%); gypenoside (2.14%) and marker saponin (1.63%).

### 2.4. Testing Sessions

Dietary intake was controlled for the week prior to each SS and TtE session via the provision of all food for the participants in an energy balanced diet as previously indicated. Participants attended the laboratory the morning after an overnight fast. After the 60 min of SS exercise, each participant had 5 min of active recovery (30 W) and then performed the TtE test as detailed earlier. Following the TtE test, participants performed a short cool down and then they consumed their dietary controlled breakfast and their allocated supplement. Participants then continued on their controlled diets and allocated supplements until the day of the 20 km TT.

Two days following the SS + TtE session, participants arrived after an overnight fast to undertake a 20 km TT on a Velotron™ cycle ergometer (SRAM LLC, Chicago, IL, USA). Following the TT, participants undertook a 4-week washout period where no interventions were undertaken. Participants were then crossed over to the alternative supplement for a 4-week period prior to the completion of the testing sessions as detailed above.

### 2.5. Muscle Analyses

Muscle samples were obtained from the vastus lateralis under local anesthesia (xylocaine 1%) utilizing a Bergström needle with suction as previously described [26]. This muscle is well characterised in terms of the consistency of fibre types and is extensively used as the main site for muscle biopsy studies in human exercise trials. There is some variation between men and women, which is another reason why we chose to recruit only young men for this trial [27]. Muscle biopsies were taken at rest (0 min), at the midpoint (30 min), and at the conclusion of the 60 min SS exercise bout. The majority of the muscle biopsies (50–100 mg) were immediately frozen in liquid nitrogen for subsequent gene and protein analyses. The remaining portions (~10–20 mg) of fresh muscle tissue collected at the rest and 60 min time points were assessed for electron transport system respiration and oxidative phosphorylation, measured using the Oxygraph O2k high-resolution respirometer (Oroboros Instruments, Innsbruck, Austria) via a substrate, uncoupler, inhibitor titration (SUIT) protocol at 37 °C in mitochondrial respiration medium 05 while stirring at 750 rpm, as previously described [28].

### 2.6. Blood Analyses

Plasma and serum samples were collected at rest prior to the muscle biopsy collection and SS exercise bout, centrifuged and then stored at −80 °C. Serum samples were analyzed for total cholesterol, high-density lipoprotein (HDL)-cholesterol, low-density lipoprotein (LDL)-cholesterol, and total triglycerides by a commercial laboratory. Plasma samples were collected for the determination of leptin and adiponectin.

Leptin levels were measured using a human leptin ELISA kit with recombinant human leptin as a standard, according to manufacturer’s instructions (Abcam, Melbourne, VIC, Australia). Briefly, 100 µL of the plasma, diluted in sample diluent, and each standard was added to the well and incubated for 2.5 h at room temperature with gentle shaking. Following this step, the plate was incubated with 100 μL of biotinylated leptin detection antibody for 1 h, 100 μL of horseradish peroxidase (HRP)–streptavidin solution for 45 min, and 100 μL of tetramethylbenzidine (TMB) one-step substrate reagent for 30 min at room temperature in the dark with gentle shaking. Stop Solution (50 μL) was added to each well, and absorbance was read at 450 nm immediately with a SpectraMax i3x Multi-Mode Microplate Reader (Molecular Devices, San Jose, CA, USA). The plate was washed three times between each step with 300 μL of washing buffer. A standard curve was generated, and leptin content for each sample was calculated.

AMPK can be stimulated in skeletal muscle via adiponectin [29,30]. As such, total adiponectin was measured using a Human Adiponectin SimpleStep ELISA^®^ Kit according to the manufacturer’s instructions (Abcam, Melbourne, VIC, Australia). Briefly, plasma samples were diluted with sample diluent NS and 1× denaturant. Standards or plasma samples were then added into the specified wells. Then, 50 μL of the antibody cocktail was added into each well and incubated for 1 h at room temperature with shaking. After washing four times with wash buffer, 100 μL of TMB substrate was added to each well and incubate for 10 min in the dark on a plate shaker. The reaction was stopped with Stop Solution, and optical density was determined at 450 nm with a microplate reader (SpectraMax i3x Multi-Mode Microplate Reader). Adiponectin content for each sample was calculated based on a standard curve.

### 2.7. RNA Isolation and Reverse Transcription

Total RNA was extracted from frozen skeletal muscle samples utilizing a TRIzol-based method according to the manufacturer’s instructions and as previously described [31]. RNA was then reverse transcribed into cDNA using the iScript^TM^ cDNA Synthesis Kit according to the manufacturer’s instructions (Bio-Rad Laboratories Pty Ltd., Gladesville, NSW, Australia). Subsequently, cDNA was stored at −20 °C until further analysis.

Real-time polymerase chain reaction (PCR) analysis of human skeletal muscle.

A panel of mRNA sequences was selected to investigate the effect of *G. Pentaphyllum* on markers of fat and carbohydrate metabolism, mitochondrial biogenesis, and function in the context of exercising muscle (Table 1). Oligonucleotide primers were designed using the Oligoperfect™ Suite (Thermo Fisher Scientific, Waltham, MA, USA) and were purchased from Integrated DNA Technologies, Inc. (1710 Commercial Park, Coralville, IA, USA). Selective gene homology for genes of interest was confirmed via BLAST (Basic Local Alignment Search Tool, National Centre for Biotechnology Information, Bethesda, MD) to determine homologous binding to the target mRNA sequence. The primer sequences for the genes of interest are shown in Table 1.

To quantify mRNA expression in human skeletal muscle, real-time PCR was conducted using SsoAdvanced^TM^ Universal SYBR Green Supermix (Bio-Rad Laboratories, Hercules, CA, USA) and QuantStudio^TM^ 7 Flex Real-Time PCR Instrument (Applied Biosystems, Waltham, MA, USA). The real-time PCR cycling parameters were as follows: initial denaturation and enzyme activation at 50 °C for 2 min and 95 °C for 10 min, followed by 40 cycles of denaturation at 95 °C for 15 s, annealing and extension at 60 °C for 1 min. Relative changes in mRNA abundance was normalized to the average of two housekeeping genes (cyclophilin and glyceraldehydes-3-phosphate dehydrogenase (GAPDH)), then quantified using the 2^−ΔΔCT^ method [32].

### 2.8. Protein Analyses

The content of specific proteins involved in energy production in human skeletal muscle was measured by western blot analysis. Muscle was lysed in buffer that contained 20 μL of Radioimmunoprecipitation assay buffer (Cell Signaling Technology, Danvers, MA, USA) for every 1 mg of muscle sample and 3 inhibitors (Protease Inhibitor Cocktail, Phosphatase Inhibitor Cocktail 2 and Phosphatase Inhibitor Cocktail 3, Sigma-Aldrich, St. Louis, MO, USA) at a proportion of 2% in the total mixture of the protein extract. Muscle samples were homogenized using a Qiagen TissueLyser II (Qiagen, Hilden, Germany). Samples were spun at 15,000× *g* for 10 min at 4 °C, and supernatant was collected. After the determination of protein concentration using Bio-Rad Protein Assay Dye Reagent Concentrate (Bio-Rad Laboratories, Hercules, CA, USA) based on the Bradford method [33], lysates were mixed with Laemmli sample buffer (Bio-Rad Laboratories, Hercules, CA, USA), and 35 µg of protein was loaded into 4–20% Criterion™ TGX Stain-Free™ Protein Gel (Bio-Rad Laboratories, Hercules, CA, USA). After electrophoresis, gels were activated with methanol for 1 min. Then, gels were transferred to TransBlot Turbo Midi-size low-fluorescence polyvinylidene fluoride (LF PVDF) membranes (Bio-Rad Laboratories, Hercules, CA, USA) with western blot transfer buffer including 25 mM Tris, 192 mM glycine, 0.01% SDS, and 20% methanol for 90 min on ice. After transfer, membranes were imaged for total protein loading before rinsing briefly in distilled water. Membranes were then blocked with 5% skim milk or 5% bovine serum albumin (BSA) for 1 h, washed 3 times (10 min each wash) with 25 mM Tris × HCl, 140 mM NaCl, 2.5 mM KCl (pH = 7.5), and 0.1% Tween 20 solution (TBST) and then incubated with a primary antibody diluted in TBST (1:1000) overnight at 4 °C on a shaker. Membranes were then washed 3 times with TBST and incubated for 1 h at room temperature with a secondary antibody goat anti-rabbit IgG H&L HRP diluted in 5% skim milk or 5% BSA (1:10,000). Proteins were detected via electrochemiluminescence (Bio-Rad Laboratories, Hercules, CA, USA and Thermo Fisher Scientific, Waltham, MA, USA) and quantified using a ChemiDoc™ MP imaging system (Bio-Rad Laboratories, Hercules, CA, USA). The band density data for each target protein was normalised to total protein content for each lane and to the internal standard loaded in each gel using the stain-free method as described previously [34,35]. Samples for each participant were run on the same gel.

All primary antibodies were purchased from Cell Signaling Technology (Danvers, MA, USA), including 5′ AMP-activated protein kinase alpha (AMPK-α) (2532), phospho-AMPKThr172 (2535), acetyl-CoA carboxylase (ACC-α; 3662), phospho-ACCSer79 (3661), and PGC-1α (2178). A secondary antibody (Anti-Rabbit IgG (Goat), HRP-Labeled) was purchased from PerkinElmer (Melbourne, Australia). The volume density of each target band was normalized to total protein loaded into each lane using stain-free technology (Bio-Rad Laboratories, Hercules, CA, USA). After protein loading normalization, each phosphoprotein was then normalized to its respective total protein. Analysis of the western blots was performed using the Image Lab software 6.0.1 (Bio-Rad Laboratories, Hercules, CA, USA). The content of each protein was quantified using the Image J software 1.53 (National Institute of Health, Bethesda, MD, USA).

### 2.9. Statistical Analysis

GraphPad Prism Software 8.4.3 (GraphPad Software, Inc., La Jolla, CA, USA) was utilized for statistical analysis. All results were expressed as mean ± standard error of the mean (SEM) for each measurement (*n* = 13–16). Paired *t*-test or paired *t*-test with Wilcoxon correction was used to analyze significant differences in the characteristics of participants, 20 km TT performance and TtE between placebo and supplementation with *G. pentaphyllum*. A repeated-measures two-way ANOVA with Geisser–Greenhouse correction and uncorrected Fisher’s LSD was performed to analyze the significant differences in the changes of mitochondrial respiration after the exercise, blood glucose measurements during steady state exercise, the mRNA expression of genes, protein expression, leptin content and adiponectin content amongst treatments. Values greater than 3 standard deviations from the mean were considered outliers and excluded from analysis. *p* < 0.05 was considered to indicate a significant difference.

## 3. Results

There was no significant difference in body weight or power output during the TtE test or SS test. Resting plasma glucose and leptin concentrations were lower and HDL cholesterol levels were higher following four weeks of supplementation with *G. pentaphyllum,* there were no other differences in blood cholesterol or triglyceride levels observed (Table 2).

**Figure 2:**
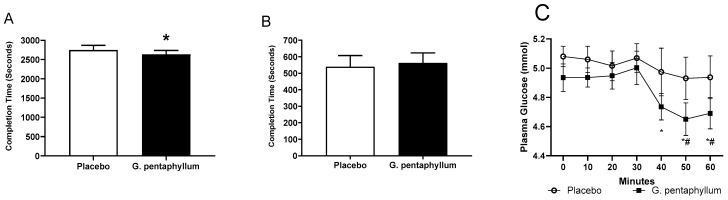
Performance trial and glucose response. The completion time (seconds) for 20 km time trial (**A**), time to exhaustion test (**B**), and plasma glucose response during the 60 min steady-state exercise prior to the time to exhaustion test (**C**). * *p* < 0.05 compared to placebo, # *p* < 0.05 compared to 0 min following *G. pentaphyllum*. Data displayed as means ± SEM. *n* = 15–16.

The 20 km TT was completed faster (112 ± 61 s) following *G. pentaphyllum* supplementation compared to placebo (2747 ± 121 vs. 2634 ± 101 s; Figure 2A, *p* < 0.05). However, there was no difference in the TtE test comparing *G. pentaphyllum* to placebo (Figure 2B). There was no difference in the VO_2_ data (28.0 ± 2.6 vs. 27.7 ± 2.5 mL·kg^−1^·min^−1^, corresponding to 67.3 ± 3.9 vs. 63.7 ± 3.5% of VO_2peak_, for placebo and *G. pentaphyllum* supplementation, respectively) during the steady state exercises after the supplementation period. Similarly, there was no difference in the respiratory exchange ratio (RER) data (0.96 ± 0.02 vs. 0.97 ± 0.02, corresponding to 87.5 ± 2.0 vs. 88.5 ± 2.8% of peak RER, for placebo and *G. pentaphyllum* supplementation, respectively) during the steady state exercises after the supplementation period. During the steady state exercise, there was a drop in plasma glucose levels after 40 min of exercise compared to placebo following *G. pentaphyllum* supplementation and after 50 min of exercise compared with the 0 min for *G. pentaphyllum* supplementation only (Figure 2C, *p* < 0.05).

**Figure 3:**
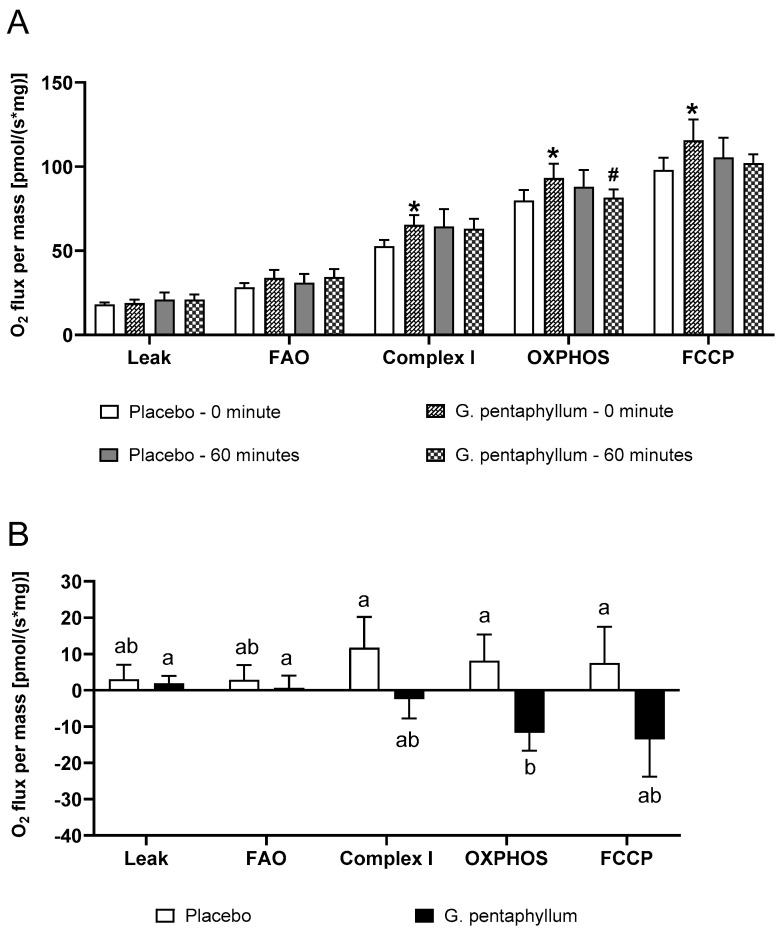
Effects of *G. pentaphyllum* supplementation on mitochondria. The effects of 60 min exercise on OXPHOS complex activities from skeletal muscle mitochondria (**A**) and the changes in mitochondrial respiration comparing rest (0 min) to after exercise (**B**) following four weeks of placebo or *G. pentaphyllum* supplementation. *n* = 9–11. * *p* < 0.05 compared to 0 min placebo. ^#^ *p* < 0.05 compared to 0 min *G. pentaphyllum* supplementation. Different letters indicate a significant difference between supplementation, *p* < 0.05.

While 15 participants completed both of the interventions, mitochondrial function in a total of 7 muscle samples could not be measured due to either damaged muscle fibers or equipment error, leaving paired sample numbers of 11 at rest and 9 matched resting (0 min) and post-exercise (60 min) samples. At rest, a higher oxygen flux per mass of fresh muscle was observed after *G. pentaphyllum* supplementation when compared to placebo at Complex I (oxidative phosphorylation (OXPHOS) capacity through Complex I), OXPHOS (maximal respiratory capacity in the respirometer chamber) and carbonyl cyanide 4-(trifluoromethoxy)phenylhydrazone (FCCP) (measurement of electron transport system (ETS) capacity) (Figure 3A, *p* < 0.05). There was, however, a reduction in maximal OXPHOS capacity following exercise while consuming *G. pentaphyllum* (Figure 3A, *p* < 0.05).

When comparing the change in O_2_ flux in paired resting (0 min) and post exercise (60 min) samples, a reduction in maximal OXPHOS capacity was observed while consuming *G. pentaphyllum* compared to placebo (Figure 3B, *p* < 0.05). Following exercise, there was no change in mitochondrial respiration compared to 0 min measurements after placebo supplementation (Figure 3A,B).

**Figure 4:**
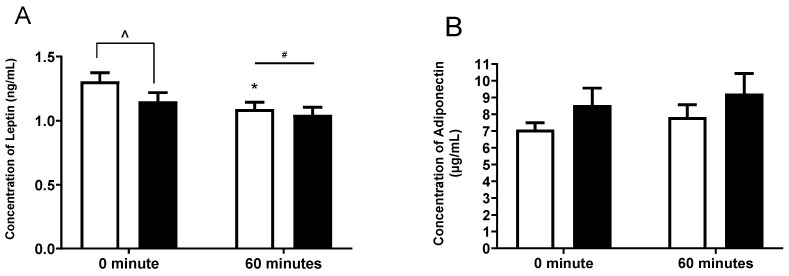
Effects of *G. pentaphyllum* supplementation on leptin and adiponectin. Determination of plasma leptin (**A**) and adiponectin (**B**) following four weeks treatment with *G. pentaphyllum* (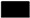 n = 15) compared to placebo (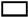 *n* = 15 and 60 min of steady state exercise. Data were expressed as mean ± SEM. * *p* < 0.05 compared to 0 min of the respective treatment. ^^^ *p* < 0.05 comparing treatments at the respective time point. ^#^ *p* < 0.05 time effect of the exercise irrespective of treatment.

The levels of leptin and adiponectin were compared following four weeks of placebo or *G. pentaphyllum* supplementation at rest (0 min) and following exercise (60 min steady state). Leptin levels were higher at rest in placebo when compared to *G. pentaphyllum* and this decreased after 60 min of exercise (Figure 4A, *p* < 0.05), and a non-significant trend to decrease was observed following *G. pentaphyllum* supplementation (Figure 4A, *p* = 0.079). There was also a treatment and an effect of exercise on leptin, with levels being overall higher in placebo and being lower following 60 min of exercise irrespective of supplementation (Figure 4A, *p* < 0.05). There was no difference in the levels of adiponectin following 60 min of exercise (Figure 4B) with either supplement. However, there was a non-significant trend for adiponectin to be elevated at rest and following exercise in the *G. pentaphyllum* treatment (Figure 4B, *p* = 0.08 and *p* = 0.07 respectively).

**Figure 5:**
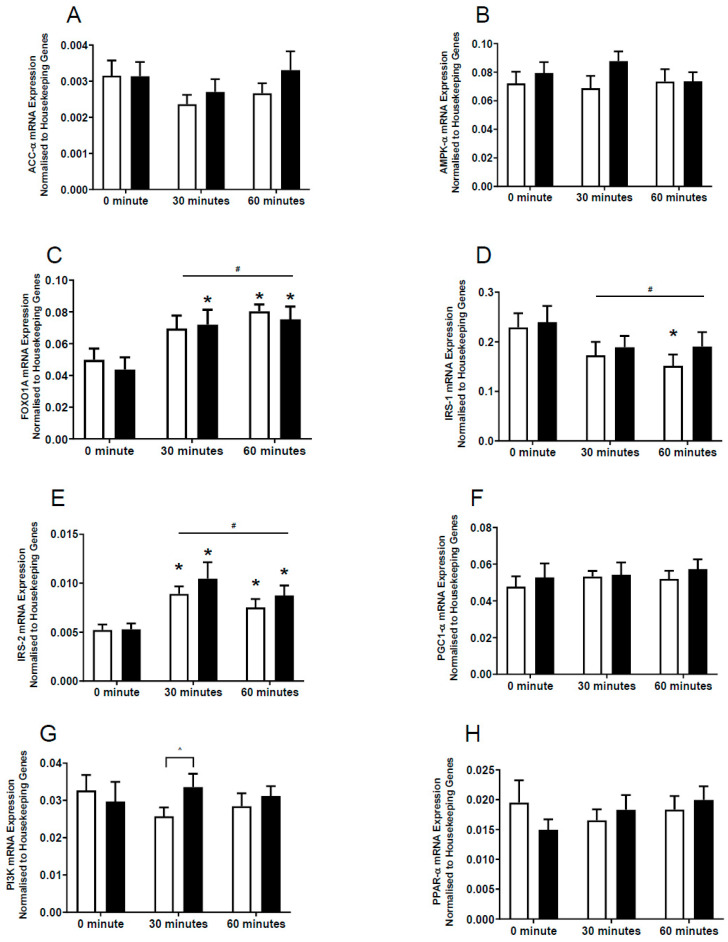
Effects of *G. pentaphyllum* supplementation on mRNA expression. The expression of (**A**) ACC-α; (**B**) AMPK-α; (**C**) FOXO1A; (**D**) IRS-1; (**E**) IRS-2; (**F**) PGC1-α; (**G**) PI3K; and (**H**) PPAR-α in skeletal muscle obtained from participants following *G. pentaphyllum* supplementation and exercise. Participants were treated with placebo (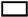 *n* = 14–15) and *G. pentaphyllum* (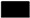 *n* = 14–15) with 0, 30, and 60 min exercise. All genes were normalized to the average of two housekeeping genes (cyclophilin and glyceraldehydes-3-phosphate dehydrogenase (GAPDH)). Data were expressed as mean ± SEM. * *p* < 0.05 compared to 0 min of the respective treatment. ^#^ *p* < 0.05 time effect of the exercise irrespective of treatment. ^ *p* < 0.05 comparing treatments at the same timepoint.

There were no significant changes in the mRNA expression of *ACC-α*, *AMPK-α*, *PGC1-α*, or peroxisome-proliferator-activated receptor alpha (*PPAR-α*) in human skeletal muscle either 30 min or 60 min after exercise following either supplement (Figure 5A,B,F,H). However, a higher mRNA expression of Forkhead box protein O1 (*FOXO1A*) was found following 30 and 60 min of exercise after *G. pentaphyllum* supplementation but only at 60 min in the placebo (Figure 5C; *p* < 0.05). A lower mRNA expression of insulin receptor substrate 1 (*IRS-1*) was observed following 60 min of exercise in placebo, compared to the resting values (Figure 5D; *p* < 0.05). On the other hand, a higher level of mRNA expression of *IRS-2* was observed in both placebo and *G. pentaphyllum* 30 and 60 min after exercise when compared to the resting values (Figure 5E; *p* < 0.05). Following 30 min of exercise, the *G. pentaphyllum* treatment had a higher mRNA expression of *PI3K* compared to placebo (Figure 5G; *p* < 0.05), which demonstrated a non-significant trend to decrease compared to rest (Figure 5G; *p* = 0.07).

A time effect was also observed as exercise resulted in higher mRNA expression of *FOXO1A* and *IRS-2* but lower *IRS-1* mRNA expression irrespective of supplement use (Figure 5C–E; *p* < 0.05).

**Figure 6:**
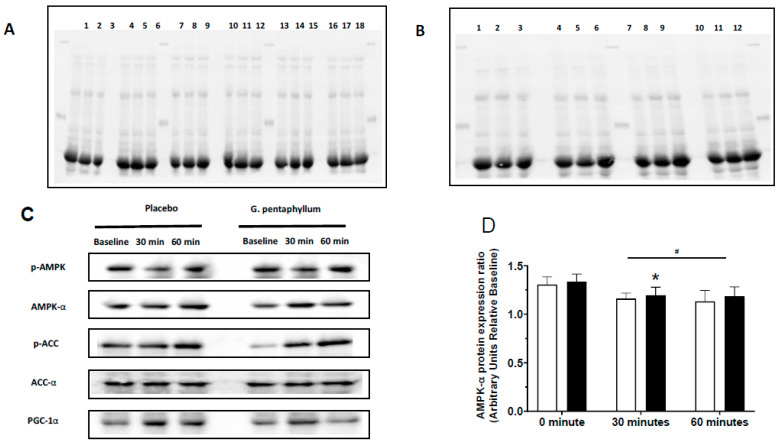

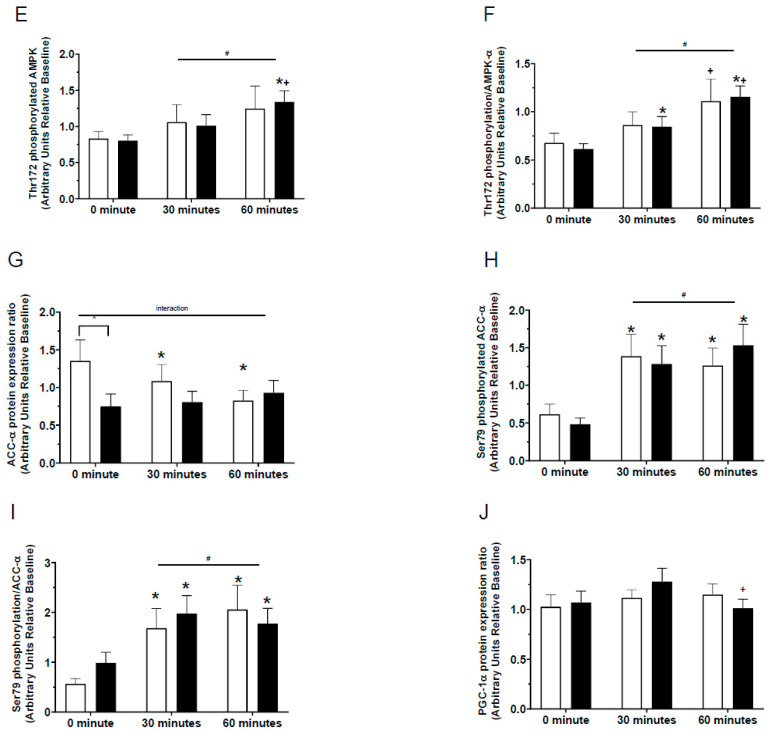
AMPK signaling pathway. AMPK signaling in protein expression in skeletal muscle obtained from participants following 4 weeks supplementation and exercise. (**A**) Full-length western blots (stain free membrane): AMPK-α—0 min, 30, and 60 min exercise with placebo (lanes 1–3) or *G. pentaphyllum* supplementation (lanes 4–6); PGC-1α–placebo (lanes 7–9) or *G. pentaphyllum* supplementation (lanes 10–12): ACC-α–placebo (lanes 13–15) or *G. pentaphyllum* supplementation (lanes 16–18). (**B**) Full-length western blots (stain free membrane): phospho-AMPK Thr172; 0 min, 30, and 60 min exercise with placebo (lanes 1–3) or *G. pentaphyllum* supplementation (lanes 4–6); phospho-ACC Ser79–placebo (lanes 7–9) or *G. pentaphyllum* supplementation (lanes 10–12). (**C**) Representative western blot and (**D**–**J**) quantification of AMPK-α, phospho-AMPKThr172, AMPK-α phosphorylation relative to total AMPK expression (ratio of p-AMPK to total AMPK), ACC-α, phospho-ACCSer79, ACC-α phosphorylation relative to total ACC expression (ratio of p-ACC to ACC-α) and PGC-1α. Participants were treated with placebo (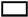 n = 13–14) and *G. pentaphyllum* supplementation (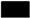 *n* = 13–14) with 30 and 60 munities exercise. Error bars represent the means ± SEM; * *p* < 0.05 compared to 0 min of the respective supplement. ^^^ *p* < 0.05 comparing treatments at the respective time. ^+^ *p* < 0.05 compared to 30 min of the respective supplement. ^#^ *p* < 0.05 time effect of the exercise irrespective of supplementation.

A lower total protein content of AMPK-α was observed in skeletal muscle following 30 min exercise with the *G. pentaphyllum* treatment, compared with 0 min (Figure 6D; *p* < 0.05). A non-significant trend was also observed at this timepoint for lower AMPK-α content in the placebo compared to *G. pentaphyllum* (Figure 6D; *p* = 0.057). A time effect was also observed, as exercise resulted in a lower AMPK-α protein irrespective of supplement (Figure 6D; *p* < 0.05).

There was a significant increase in AMPK Thr172 phosphorylation levels after 60 min exercise compared to 0 min and 30 min of exercise, with *G. pentaphyllum* supplementation (Figure 6E; *p* < 0.05). No difference in AMPK Thr172 phosphorylation was observed at any time point compared to 0 min following placebo (Figure 6E). A time effect was also observed, as exercise resulted in higher AMPK Thr172 phosphorylation levels irrespective of supplement (Figure 6E; *p* < 0.05).

Levels of AMPK Thr172 phosphorylation relative to total AMPK demonstrated an increase following 30 min of exercise, which was further increased after 60 min of exercise, with *G. pentaphyllum* supplementation (Figure 6F; *p* < 0.05). While the ratio of AMPK Thr172 phosphorylation levels to total AMPK only increased after 60 min exercise compared with 30 min of exercise following Placebo (Figure 6F; *p* < 0.05), with a non-significant trend compared to 0 min (Figure 6F; *p* = 0.074).

Resting data for ACC-α were different between treatments, with ACC-α being lower following *G. pentaphyllum* supplementation (Figure 6G; *p* < 0.05). There was a significant decrease in ACC-α levels after 30 and 60 min of exercise compared to 0 min following placebo treatment (Figure 6G; *p* < 0.05), with a non-significant trend for a further reduction from 30 to 60 min (Figure 6G; *p* = 0.053). No changes were observed with *G. pentaphyllum* supplementation, with a non-significant trend for an increase in ACC-α only following 60 min of exercise (Figure 6G; *p* = 0.08). An interaction was also observed between treatments and time (Figure 6G; *p* < 0.05).

Phospho-ACC at Ser79 (Figure 6H) and phospho-ACC at Ser79 relative to total ACC (Figure 6I) were greater after 30 and 60 min of exercise compared to 0 min in both placebo and *G. pentaphyllum* (*p* < 0.05), along with a time effect (*p* < 0.05).

The protein expression of PGC-1α level decreased after 60 min exercise compared with 30 min exercise following *G. pentaphyllum* supplementation (Figure 6J; *p* < 0.05). No other differences were observed in PGC-1α level due to supplementation or exercise.

## 4. Discussion

*G. pentaphyllum* is an herb that contains many bioactive components which have been suggested as beneficial in the treatment of various diseases, including obesity [5,9]. The bioactive saponins found in *G. pentaphyllum* have been demonstrated to increase the phosphorylation of AMPK in L6 myotube cells [12] and to enhance glucose uptake [11]. An increase in AMPK activation has been shown to enhance exercise performance in mice [16]. In the current study, *G. pentaphyllum* supplementation, provided in a randomized placebo-controlled double-blind crossover trial to young healthy males, resulted in an improved 20 km TT performance, increased mitochondrial oxidative capacity and HDL cholesterol levels, a decrease in resting plasma leptin, and a decrease in glucose levels at rest and during steady state exercise. However, in contrast to no change in mRNA expression of *AMPK-α* or *ACC-α* in either treatment, a decrease in PGC-1α protein after exercise and a significant increase in AMPK activity were found following *G. pentaphyllum* supplementation.

In our study, *G. pentaphyllum* supplementation decreased plasma leptin level at rest despite participants’ healthy weights at the start of the study and no change in weight observed throughout the trial. Leptin is a hormone responsible for regulating appetite and body weight [36]. Moreover, previous studies reported a positive relationship between leptin levels and body fat [37,38], suggesting that leptin may play an important role in obesity. This result is somewhat consistent with previous studies, which reported the effectiveness of *G. pentaphyllum* on weight loss with good tolerability [5,9]. *G. pentaphyllum* extract significantly reduced body weight and fat mass in obese individuals during 12-weeks of supplementation at doses of 450 mg/day [5]. Similarly, *G. pentaphyllum* significantly reduced body weight in overweight men and women after 16 weeks of supplementation [9]. In both previous studies [5,9], participants were overweight, and *G. pentaphyllum* supplementation was given for 12 weeks and 16 weeks, respectively, in contrast to our study, in which supplementation was given for 4 weeks and the participants were within the healthy weight range. Despite the maintenance of a healthy weight and the provision of a standardized diet in the week prior to testing, *G. pentaphyllum* decreased leptin levels, which—together with the trend for an increase in adiponectin level—may suggest an effect of the supplement on hormonal secretion from adipose tissue and warrants further investigation.

In addition to the reduction in plasma leptin levels, the current study also observed a reduction in fasting plasma glucose levels and lower glucose levels during exercise. However, it has been found in the Zucker rat model that *G. pentaphyllum* did not significantly reduce glucose levels at 120 min in the lean strain in contrast to the 20% decrease in obese Zucker rats after sucrose-induced hyperglycemia [39]. It was also demonstrated in overweight men and women with normal blood glucose levels that *G. pentaphyllum* did not alter blood glucose levels [9]. While the discrepancy between the current study and other research in situations of normoglycemia is unclear, it has been demonstrated in individuals with type II diabetes that the use of *G. pentaphyllum* decreases blood glucose levels compared to placebo [40,41,42].

This current study is the first double-blind, placebo-controlled study to examine the effects of *G. pentaphyllum* supplementation (4 weeks) on exercise performance in untrained healthy males. In the current study, *G. pentaphyllum* supplementation improved 20 km TT performance by 112 ± 61 s or approximately 4%. However, no change in TtE was observed, which is different from a previous study in mice in which daily *G. pentaphyllum* supplementation for 1 week increased TtE during a forced swim test [43] and a 6 week training study in mice [44]. In addition to the role of *G. pentaphyllum* in scavenging reactive oxygen species produced excessively during exhaustive exercises, it was also speculated that the increased glycogen level in muscles may also be a reason for improved performance in tests in mice [43]. Furthermore, rats showed a significant increase in swimming TtE after 30 days *G. pentaphyllum* supplementation with a dose of 400 mg/day [45]. These animal studies, in addition to our human study, suggest that *G. pentaphyllum* supplementation may enhance the endurance capacity of muscles via multiple mechanisms.

Another possible mechanism for improved endurance performance by *G. pentaphyllum* is via the activation of AMPK, as extracts of this supplement have been previously demonstrated to increase phosphorylation of AMPK in cell models [12]. The increased activation of AMPK is a potent stimulus for exercise performance, as 5-aminoimidazole-4-carboxamide ribonucleotide (AICAR)—an analogue of adenosine monophosphate, at a dose of 500 mg/kg/day—enhanced running speed by 23% and increased distance by 44% in sedentary mice [46]. AMPK activation has multiple effects on the molecular characteristics of skeletal muscle, which might also contribute in other ways to the enhanced exercise capacity. ACC synthesizes malonyl CoA, which in turn inhibits fatty acid (FA) oxidation in skeletal muscle mitochondria [47]. Activated AMPK inhibits ACC through phosphorylation [47]. Therefore, AMPK-induced reduction in ACC activity may promote FA oxidation in skeletal muscles by reducing malonyl CoA level in skeletal muscle [47]. In partial agreement with the effects of *G. pentaphyllum* on AMPK in cell culture, we found a lower total ACC protein level at rest and an increase in AMPK phosphorylation during exercise following *G. pentaphyllum* supplementation even though we did not observe any differences in the gene expression of ACC or AMPK. These results suggest that the alteration in AMPK signaling via *G. pentaphyllum* may be at least partially responsible for the observed improvement in exercise performance in the current study.

AMPK also regulates the activity and phosphorylation of PGC-1α [48]. PGC-1α regulates the transcription factors that are associated with energy metabolism, anti-oxidative stress, and mitochondrial biogenesis [44]. A previous study reported that *G. pentaphyllum* significantly increased *PGC-1α* expression, which upregulates mitochondrial biogenesis by activating mitochondrial genes in the nucleus [44]. However, there was no difference in *PGC-1α* mRNA expression at rest or during exercise in our study. *G. pentaphyllum* supplementation also resulted in a decreased protein content of PGC-1α after 60 min of exercise as compared to 30 min exercise, however, this was not different from resting values or placebo treatment. In our study, despite no alteration in PGC-1α at rest after *G. pentaphyllum* supplementation, we observed higher oxygen flux at Complex I, OXPHOS, and FCCP at rest after *G. pentaphyllum* supplementation when compared to placebo, suggesting increased mitochondrial function. Mitochondrial content/function is known to be associated with endurance exercise capacity [49]. Specifically, increased mitochondrial function after exercise is associated with improved 20 km TT [50]. Mitochondria-targeted antioxidant supplementation even improved TT performance in trained male cyclists [51]. While the decrease in PGC-1α following exercise would be unlikely to have an immediate effect on mitochondrial respiration, we observed a reduction in maximal OXPHOS capacity after exercise during *G. pentaphyllum* supplementation compared to placebo. Previous animal studies reported an increase in mitochondrial OXPHOS after a single session of exercise [52], while a human study reported increased oxygen flux at Complex I after a session of continuous endurance exercise but not in OXPHOS or FCCP [53]. Another study even reported decreased OXPHOS in human skeletal muscle after acute high intensity exercise [54]. Thus, in light of the increase in OXPHOS at rest but decrease after exercise, the impact of *G. pentaphyllum* supplementation on OXPHOS capacity following a period of exercise training warrants further investigation.

In our study, *G. pentaphyllum* supplementation increased mRNA expression of *P13K* and *FOXO1A* following 30 min of exercise, which was earlier than the changes observed at 60 min following placebo. It has been reported that *P13K* may increase insulin induced glucose uptake in muscles [4]. Furthermore, increased expression of *FOXO1A* stimulates expression of lipoprotein lipase in muscle cells, thus enhancing FA availability to muscles [55]. These changes, along with the changes observed in resting and exercise blood glucose levels, improvements in mitochondrial oxidative capacity and AMPK pathways, suggest that *G. pentaphyllum* supplementation may affect skeletal muscle metabolism via multiple mechanisms.

## 5. Conclusions

This study demonstrated that *G. pentaphyllum* supplementation improved mitochondrial respiration, lowered muscle total ACC, and plasma leptin levels at rest and improved performance in a 20 km TT by approximately 4% in healthy males. These results add significantly to previous research, which demonstrated the body weight management effects of *G. pentaphyllum* in obese participants [5,9], and suggest *G. pentaphyllum*’s impact on skeletal muscle as a possible key site of action. The current study had a number of limitations, including that only healthy men were recruited, the length of the intervention, when muscle and blood samples were collected, the type of exercise undertaken, and the dosage of supplement chosen. Future studies are required to further understand the effects of *G. pentaphyllum* supplementation on skeletal muscle and exercise performance, along with leptin and adiponectin level, in healthy trained individuals as well as individuals with obesity and diabetes. However, this is the first study to demonstrate the beneficial effect of *G. pentaphyllum* supplementation on exercise performance in healthy males, which corresponded with improved mitochondrial respiration, altered AMPK and ACC, and decreased plasma leptin and glucose levels at rest and decreased glucose levels during exercise.

## Figures and Tables

**Table 1:** Human primer sequences used for Real-Time PCR analysis of human skeletal muscle.

Gene	Accession Number	Sequence
ACC-α	NM_198836.2	Forward (5′–3′) CTGGAGCCCTCAACAAAGTC Reverse (5′–3′) CCAGTGCAGGACAGTGAAAA
AMPK-α	XM_016919269	Forward (5′–3′) AACTGCAGAGAGCCATTCACTTT Reverse (5′–3′) GGTGAAACTGAAGACAATGTGCTT
Cyclophilin	XM_024747056	Forward (5′–3′) CATCTGCACTGCCAAGACTGA Reverse (5′–3′) TTCATGCCTTCTTTCACTTTGC
FoxO1	XM_522749	Forward (5′–3′) TCATGGATGGAGATACATTGGATT Reverse (5′–3′) TCCTGCTGTCAGACAATCTGAAG
GAPDH	XM_024733357	Forward (5′–3′) CAA CGA CCA CTT TGT CAA GC Reverse (5′–3′) TTA CTC CTT GGA GGC CAT GT
IRS-1	NM_000208	Forward (5′–3′) GTTTCCAGAAGCAGCCAGAG Reverse (5′–3′) TGAAATGGATGCATCGTACC
IRS-2	NM_003749.2	Forward (5′–3′) ACGCCAGCATTGACTTCTTGT Reverse (5′–3′) TGACATGTGACATCCTGGTGATAA
PGC1-α	NM_013261.4	Forward (5′–3′) CAAGCCAAACCAACAACTTTATCTCT Reverse (5′–3′) CACACTTAAGGTGCGTTCAATAGTC
PI3K	NM_181504.3	Forward (5′–3′) GGAAGCAGCAACCGAAACAA Reverse (5′–3′) TTCGCCGTCCACCACTACA
PPAR-α	XM_024452253	Forward (5′–3′) GAAGCTGTCCTGGCTCAGAT Reverse (5′–3′) GGGGACCACAGGATAAGTCA

ACC-α, acetyl-CoA carboxylase alpha; AMPK-α, 5′ AMP-activated protein kinase alpha; FoxO1, forkhead box protein O1; GAPDH, glyceraldehyde 3-phosphate dehydrogenase; IRS-1, insulin receptor substrate 1; IRS-2, insulin receptor substrate 2; PGC1-α, peroxisome-proliferator-activated receptor-gamma coactivator 1-alpha; PI3K, phosphoinositide 3-kinase; PPAR-α, peroxisome-proliferator-activated receptor alpha.

**Table 2:** Participant characteristics following each treatment.

	Participants	
	Placebo	*G. pentaphyllum*
Age (years)	23.3 ± 1.1	
Height (cm)	174.4 ± 1.6	
Body Mass (kg)	69.0 ± 1.9	68.7 ± 1.8
BMI (kg/m^2^)	22.6 ± 0.5	22.6 ± 0.5
VO_2_ peak (mL·kg^−1^·min^−1^)	40.8 ± 1.7	
Peak power (Watts)	214.3 ± 11.3	
TtE power output (Watts)	161.2 ± 9.2	160.9 ± 9.3
SS power output (Watts)	103.6 ± 6.5	105.2 ± 7.2
Plasma fasting glucose (mmol/L)	5.15 ± 0.11	4.86 ± 0.07 *
Total cholesterol (mmol/L)	3.71 ± 0.18	3.76 ± 0.14
HDL (mmol/L)	1.24 ± 0.04	1.3 ± 0.05 *
LDL (mmol/L)	2.05 ± 0.18	1.98 ± 0.12
LDL/HDL	1.69 ± 0.16	1.56 ± 0.13
Total cholesterol/HDLC	3.04 ± 0.16	2.90 ± 0.16
Total triglycerides (mmol/L)	0.98 ± 0.11	1.04 ± 0.16
Adiponectin (µg/mL)	7.09 ± 0.42	8.55 ± 1.02
Leptin (ng/mL)	1.31 ± 0.07	1.15 ± 0.07 *

LDL: low-density lipoprotein–cholesterol; HDL: high-density lipoprotein–cholesterol; TtE: time to exhaustion test; SS: steady state. Values are expressed as means ± SEM. *n* = 16. * *p* < 0.05 compared to placebo.

## Data Availability

The data presented in this study are available on request from the corresponding author. The data are not publicly available due to ethical restrictions.

## References

[1] Lokman E.F., Gu H.F., Wan Mohamud W.N., Ostenson C.G. (2015). Evaluation of Antidiabetic Effects of the Traditional Medicinal Plant *Gynostemma pentaphyllum* and the Possible Mechanisms of Insulin Release. Evid. Based Complement. Altern. Med..

[2] Li Y., Lin W., Huang J., Xie Y., Ma W. (2016). Anti-cancer effects of *Gynostemma pentaphyllum* (Thunb.) Makino (Jiaogulan). Chin. Med..

[3] Chiranthanut N., Teekachunhatean S., Panthong A., Khonsung P., Kanjanapothi D., Lertprasertsuk N. (2013). Toxicity evaluation of standardized extract of *Gynostemma pentaphyllum* Makino. J. Ethnopharmacol..

[4] Huang T.H., Razmovski-Naumovski V., Salam N.K., Duke R.K., Tran V.H., Duke C.C., Roufogalis B.D. (2005). A novel LXR-alpha activator identified from the natural product *Gynostemma pentaphyllum*. Biochem. Pharmacol..

[5] Park S.H., Huh T.L., Kim S.Y., Oh M.R., Tirupathi Pichiah P.B., Chae S.W., Cha Y.S. (2014). Antiobesity effect of *Gynostemma pentaphyllum* extract (actiponin): A randomized, double-blind, placebo-controlled trial. Obesity.

[6] Takemoto T., Arihara S., Nakajima T., Okuhira M. (1983). Studies on the constituents of fructus Momordicae. III. Structure of mogrosides. Yakugaku Zasshi.

[7] Hung T.M., Thu C.V., Cuong T.D., Hung N.P., Kwack S.J., Huh J.I., Min B.S., Choi J.S., Lee H.K., Bae K. (2010). Dammarane-type glycosides from *Gynostemma pentaphyllum* and their effects on IL-4-induced eotaxin expression in human bronchial epithelial cells. J. Nat. Prod..

[8] Su C., Li N., Ren R., Wang Y., Su X., Lu F., Zong R., Yang L., Ma X. (2021). Progress in the Medicinal Value, Bioactive Compounds, and Pharmacological Activities of *Gynostemma pentaphyllum*. Molecules.

[9] Rao A., Clayton P., Briskey D. (2022). The effect of an orally-dosed *Gynostemma pentaphyllum* extract (ActivAMP(R)) on body composition in overweight, adult men and women: A double-blind, randomised, placebo-controlled study. J. Hum. Nutr. Diet..

[10] Xie P., Guo M., Xie J.B., Xiao M.Y., Qi Y.S., Duan Y., Li F.F., Piao X.L. (2022). Effects of heat-processed *Gynostemma pentaphyllum* on high-fat diet-fed mice of obesity and functional analysis on network pharmacology and molecular docking strategy. J. Ethnopharmacol..

[11] Lee H.S., Lim S.M., Jung J.I., Kim S.M., Lee J.K., Kim Y.H., Cha K.M., Oh T.K., Moon J.M., Kim T.Y. (2019). *Gynostemma pentaphyllum* Extract Ameliorates High-Fat Diet-Induced Obesity in C57BL/6N Mice by Upregulating SIRT1. Nutrients.

[12] Nguyen P.H., Gauhar R., Hwang S.L., Dao T.T., Park D.C., Kim J.E., Song H., Huh T.L., Oh W.K. (2011). New dammarane-type glucosides as potential activators of AMP-activated protein kinase (AMPK) from *Gynostemma pentaphyllum*. Bioorg Med. Chem..

[13] Gauhar R., Hwang S.-L., Jeong S.-S., Kim J.-E., Song H., Park D.C., Song K.-S., Kim T.Y., Oh W.K., Huh T.-L. (2012). Heat-processed *Gynostemma pentaphyllum* extract improves obesity in ob/ob mice by activating AMP-activated protein kinase. Biotechnol. Lett..

[14] Zhang B.B., Zhou G., Li C. (2009). AMPK: An emerging drug target for diabetes and the metabolic syndrome. Cell Metab..

[15] Hardie D.G. (2007). AMP-activated/SNF1 protein kinases: Conserved guardians of cellular energy. Nat. Rev. Mol. Cell Biol..

[16] Narkar V.A., Downes M., Yu R.T., Embler E., Wang Y.X., Banayo E., Mihaylova M.M., Nelson M.C., Zou Y., Juguilon H. (2008). AMPK and PPARdelta agonists are exercise mimetics. Cell.

[17] Wu S.B., Wu Y.T., Wu T.P., Wei Y.H. (2014). Role of AMPK-mediated adaptive responses in human cells with mitochondrial dysfunction to oxidative stress. Biochim. Biophys. Acta.

[18] Bergeron R., Ren J.M., Cadman K.S., Moore I.K., Perret P., Pypaert M., Young L.H., Semenkovich C.F., Shulman G.I. (2001). Chronic activation of AMP kinase results in NRF-1 activation and mitochondrial biogenesis. Am. J. Physiol. Endocrinol. Metab..

[19] Slavin M.B., Memme J.M., Oliveira A.N., Moradi N., Hood D.A. (2022). Regulatory networks coordinating mitochondrial quality control in skeletal muscle. Am. J. Physiol.-Cell Physiol..

[20] Timmers S., Konings E., Bilet L., Houtkooper R.H., van de Weijer T., Goossens G.H., Hoeks J., van der Krieken S., Ryu D., Kersten S. (2011). Calorie restriction-like effects of 30 days of resveratrol supplementation on energy metabolism and metabolic profile in obese humans. Cell Metab..

[21] Blazev R., Carl C.S., Ng Y.K., Molendijk J., Voldstedlund C.T., Zhao Y., Xiao D., Kueh A.J., Miotto P.M., Haynes V.R. (2022). Phosphoproteomics of three exercise modalities identifies canonical signaling and C18ORF25 as an AMPK substrate regulating skeletal muscle function. Cell Metab..

[22] Australian Institute of Health and Welfare (AIHW) (2003). The Active Australia Survey: A Guide and Manual for Implementation, Analysis and Reporting. http://www.aihw.gov.au.

[23] Laursen P.B., Shing C.M., Jenkins D.G. (2003). Reproducibility of a laboratory-based 40-km cycle time-trial on a stationary wind-trainer in highly trained cyclists. Int. J. Sports Med..

[24] Laudisi F., Di Fusco D., Dinallo V., Stolfi C., Di Grazia A., Marafini I., Colantoni A., Ortenzi A., Alteri C., Guerrieri F. (2019). The Food Additive Maltodextrin Promotes Endoplasmic Reticulum Stress-Driven Mucus Depletion and Exacerbates Intestinal Inflammation. Cell Mol. Gastroenterol. Hepatol..

[25] Kim T.Y., Moon J.M., Kyong S.H., Kim Y.H. (2020). Preparation Method of *Gynostemma pentaphyllum* Leaves Extract for Increasing Small Molecular Effective Saponin Contents and Decreasing Benzopyrene. U.S. Patent.

[26] Yan X., Eynon N., Papadimitriou I.D., Kuang J., Munson F., Tirosh O., O’Keefe L., Griffiths L.R., Ashton K.J., Byrne N. (2017). The gene SMART study: Method, study design, and preliminary findings. BMC Genom..

[27] Staron R.S., Hagerman F.C., Hikida R.S., Murray T.F., Hostler D.P., Crill M.T., Ragg K.E., Toma K. (2000). Fiber type composition of the vastus lateralis muscle of young men and women. J. Histochem. Cytochem..

[28] Timpani C.A., Trewin A.J., Stojanovska V., Robinson A., Goodman C.A., Nurgali K., Betik A.C., Stepto N., Hayes A., McConell G.K. (2017). Attempting to Compensate for Reduced Neuronal Nitric Oxide Synthase Protein with Nitrate Supplementation Cannot Overcome Metabolic Dysfunction but Rather Has Detrimental Effects in Dystrophin-Deficient mdx Muscle. Neurotherapeutics.

[29] McAinch A.J., Steinberg G.R., Mollica J., O’Brien P.E., Dixon J.B., Macaulay S.L., Kemp B.E., Cameron-Smith D. (2006). Differential regulation of adiponectin receptor gene expression by adiponectin and leptin in myotubes derived from obese and diabetic individuals. Obesity.

[30] Chen M.B., McAinch A.J., Macaulay S.L., Castelli L.A., O’Brien P.E., Dixon J.B., Cameron-Smith D., Kemp B.E., Steinberg G.R. (2005). Impaired activation of AMP-kinase and fatty acid oxidation by globular adiponectin in cultured human skeletal muscle of obese type 2 diabetics. J. Clin. Endocrinol. Metab..

[31] Simcocks A.C., O’Keefe L., Jenkin K.A., Cornall L.M., Grinfeld E., Mathai M.L., Hryciw D.H., McAinch A.J. (2020). The Role of Atypical Cannabinoid Ligands O-1602 and O-1918 on Skeletal Muscle Homeostasis with a Focus on Obesity. Int. J. Mol. Sci..

[32] Godfrey T.E., Kim S.H., Chavira M., Ruff D.W., Warren R.S., Gray J.W., Jensen R.H. (2000). Quantitative mRNA expression analysis from formalin-fixed, paraffin-embedded tissues using 5’ nuclease quantitative reverse transcription-polymerase chain reaction. J. Mol. Diagn..

[33] Bradford M.M. (1976). A rapid and sensitive method for the quantitation of microgram quantities of protein utilizing the principle of protein-dye binding. Anal. Biochem..

[34] Gilda J.E., Gomes A.V. (2013). Stain-Free total protein staining is a superior loading control to β-actin for Western blots. Anal. Biochem..

[35] Gürtler A., Kunz N., Gomolka M., Hornhardt S., Friedl A.A., McDonald K., Kohn J.E., Posch A. (2013). Stain-Free technology as a normalization tool in Western blot analysis. Anal. Biochem..

[36] Farr O.M., Gavrieli A., Mantzoros C.S. (2015). Leptin applications in 2015: What have we learned about leptin and obesity?. Curr Opin Endocrinol. Diabetes Obes..

[37] Izquierdo A.G., Crujeiras A.B., Casanueva F.F., Carreira M.C. (2019). Leptin, Obesity, and Leptin Resistance: Where Are We 25 Years Later?. Nutrients.

[38] Landecho M.F., Tuero C., Valenti V., Bilbao I., de la Higuera M., Fruhbeck G. (2019). Relevance of Leptin and Other Adipokines in Obesity-Associated Cardiovascular Risk. Nutrients.

[39] Megalli S., Davies N.M., Roufogalis B.D. (2006). Anti-hyperlipidemic and hypoglycemic effects of *Gynostemma pentaphyllum* in the Zucker fatty rat. J. Pharm. Pharm. Sci..

[40] Huyen V.T., Phan D.V., Thang P., Ky P.T., Hoa N.K., Ostenson C.G. (2012). Antidiabetic Effects of Add-On *Gynostemma pentaphyllum* Extract Therapy with Sulfonylureas in Type 2 Diabetic Patients. Evid. Based Complement. Altern. Med..

[41] Huyen V.T., Phan D.V., Thang P., Hoa N.K., Ostenson C.G. (2010). Antidiabetic effect of *Gynostemma pentaphyllum* tea in randomly assigned type 2 diabetic patients. Horm. Metab. Res..

[42] Huyen V.T., Phan D.V., Thang P., Hoa N.K., Ostenson C.G. (2013). *Gynostemma pentaphyllum* Tea Improves Insulin Sensitivity in Type 2 Diabetic Patients. J. Nutr. Metab..

[43] Chi A., Tang L., Zhang J., Zhang K. (2012). Chemical composition of three polysaccharides from *Gynostemma pentaphyllum* and their antioxidant activity in skeletal muscle of exercised mice. Int. J. Sport. Nutr. Exerc. Metab..

[44] Kim Y.H., Jung J.I., Jeon Y.E., Kim S.M., Hong S.H., Kim T.Y., Kim E.J. (2022). *Gynostemma pentaphyllum* extract and its active component gypenoside L improve the exercise performance of treadmill-trained mice. Nutr. Res. Pract..

[45] Lin-Na S., Yong-Xiu S. (2014). Effects of polysaccharides from *Gynostemma pentaphyllum* (Thunb.), Makino on physical fatigue. Afr. J. Tradit. Complement. Altern. Med..

[46] Pokrywka A., Cholbinski P., Kaliszewski P., Kowalczyk K., Konczak D., Zembron-Lacny A. (2014). Metabolic modulators of the exercise response: Doping control analysis of an agonist of the peroxisome proliferator-activated receptor delta (GW501516) and 5-aminoimidazole-4-carboxamide ribonucleotide (AICAR). J. Physiol. Pharmacol..

[47] Winder W.W., Holmes B.F. (2000). Insulin stimulation of glucose uptake fails to decrease palmitate oxidation in muscle if AMPK is activated. J. Appl. Physiol..

[48] McConell G.K., Ng G.P., Phillips M., Ruan Z., Macaulay S.L., Wadley G.D. (2010). Central role of nitric oxide synthase in AICAR and caffeine-induced mitochondrial biogenesis in L6 myocytes. J. Appl. Physiol..

[49] Holloszy J.O. (1967). Biochemical adaptations in muscle. Effects of exercise on mitochondrial oxygen uptake and respiratory enzyme activity in skeletal muscle. J. Biol. Chem..

[50] Granata C., Oliveira R.S., Little J.P., Renner K., Bishop D.J. (2016). Mitochondrial adaptations to high-volume exercise training are rapidly reversed after a reduction in training volume in human skeletal muscle. FASEB J. Off. Publ. Fed. Am. Soc. Exp. Biol..

[51] Broome S.C., Braakhuis A.J., Mitchell C.J., Merry T.L. (2021). Mitochondria-targeted antioxidant supplementation improves 8 km time trial performance in middle-aged trained male cyclists. J. Int. Soc. Sports Nutr..

[52] Yoo S.Z., No M.H., Heo J.W., Chang E., Park D.H., Kang J.H., Seo D.Y., Han J., Jung S.J., Hwangbo K. (2019). Effects of a single bout of exercise on mitochondria-mediated apoptotic signaling in rat cardiac and skeletal muscles. J. Exerc. Rehabil..

[53] Trewin A.J., Parker L., Shaw C.S., Hiam D.S., Garnham A., Levinger I., McConell G.K., Stepto N.K. (2018). Acute HIIE elicits similar changes in human skeletal muscle mitochondrial H_2_O_2_ release, respiration, and cell signaling as endurance exercise even with less work. Am. J. Physiol. Regul. Integr. Comp. Physiol..

[54] Layec G., Blain G.M., Rossman M.J., Park S.Y., Hart C.R., Trinity J.D., Gifford J.R., Sidhu S.K., Weavil J.C., Hureau T.J. (2018). Acute High-Intensity Exercise Impairs Skeletal Muscle Respiratory Capacity. Med. Sci. Sports Exerc..

[55] Hirano R., Igarashi O., Kondo K., Itakura H., Matsumoto A. (2001). Regulation by long-chain fatty acids of the expression of cholesteryl ester transfer protein in HepG2 cells. Lipids.

